# Inhibition of poly (ADP-ribose) polymerase and inducible nitric oxide synthase protects against ischemic myocardial damage by reduction of apoptosis

**DOI:** 10.3892/mmr.2014.2977

**Published:** 2014-11-19

**Authors:** JUAN WANG, LIN HAO, YAN WANG, WEIDONG QIN, XIN WANG, TONG ZHAO, YUSHENG LIU, LIN SHENG, YIMENG DU, MENGYUAN ZHANG, QINGHUA LU

**Affiliations:** 1 Department of Cardiovascular Medicine, The Second Hospital of Shandong University, Jinan, Shandong 250033, P.R. China; 2 Department of Anesthesiology, Provincial Hospital Affiliated to Shandong University, Jinan, Shandong 250062, P.R. China; 3 Department of Intensive Care Unit, Qilu Hospital Affiliated to Shandong University, Jinan, Shandong 250012, P.R. China

**Keywords:** myocardial infarction, poly (ADP-ribose) polymerase 1, inducible nitric oxide synthase, apoptosis, peroxynitrite

## Abstract

Myocardial infarction (MI) is defined as the deprivation of the myocardial tissue of oxygen and nutrients, resulting in the induction of inflammation and apoptosis of the cardiomyocytes. Poly (ADP-ribose) polymerase 1 (PARP1) is a nuclear enzyme closely associated with MI, that can be activated by DNA damage. Inducible nitric oxide synthase (iNOS) is a critical enzyme among the inflammatory cytokines. The present study aimed to investigate the underlying mechanism of the protective effects of PARP1 and iNOS inhibitor against MI, in rats. A total of 40 male Wistar rats were divided into four groups. The rats were anesthetized with sodium pentobarbital (50 mg/kg), and the left anterior descending coronary artery was occluded by ligation, using a 6-0 polypropylene monofilament suture, at the left atrial apex, in order to induce MI. The rats from each group received an abdominal injection of either dimethylsulfoxide (100 μl, for MI group); PARP-1 inhibitor, 3,4-dihydro-5-[4-(1-piperidinyl)butoxy]-1(2H)-isoquinolinone (DPQ; 10 mg/kg); or iNOS inhibitor, N-(1-naphthyl)ethylenediamine dihydrochloride (1400W; 10 mg/kg). The hearts were harvested from the rats after four weeks. Inhibition of PARP and iNOS activity improved heart function, as determined by serial echocardiography. The rate of apoptosis, as determined by a terminal deoxynucleotidyl-transferase-mediated dUTP nick end labeling assay, was reduced by 39.71 and 39.00% in the DPQ and 1400W groups, respectively, and this was accompanied by the downregulated expression of cleaved caspase-3 and PARP1. Effective inhibition of PARP and iNOS, by DPQ and 1400W, was detected by western blotting and immunofluorescence, and was shown to repress O_2_^−^ and nitrotyrosine levels, following MI. The present study confirmed that inhibition of PARP1 and iNOS was able to protect against ischemic myocardial damage, by reducing the levels of apoptosis.

## Introduction

Myocardial infarction (MI) is a common cardiovascular disease, and one of the leading causes of mortality, worldwide (1). MI is caused by myocardial deprivation of oxygen and nutrients, which results in the induction of the inflammatory response and apoptosis of cardiomyocytes (2). Various factors are involved in the process, including poly (ADP-ribose) polymerase 1 (PARP1).

PARP1 is a nuclear enzyme, which functions as a DNA damage sensor, and can be activated by DNA strand breaks (3). Activation of PARP1 leads to the synthesis of poly(ADP-ribose) (PAR), from nicotinamide adenine dinucleotide (NAD) and ATP, which is essential for DNA repair (4). However, excessive activation of PARP1 may result in the depletion of NAD and ATP, which leads to cellular dysfunction and eventual cell death (5). Furthermore, PARP1 is required for specific NF-κB-dependent gene activation, and acts as a transcriptional co-activator of NF-κB (6). It has been shown to regulate various key inflammatory cytokines, including monocyte chemotactic protein-1, inducible nitric oxide synthase (iNOS), and adhesion molecules, all of which are known to be regulated by NF-κB(7–9). Excessive activation of PARP1 has been shown to be associated with the pathogenesis of numerous diseases, including energetic failure and vascular collapse in shock, diabetes, cerebral ischemia, endothelial dysfunction in hypertension, atherosclerosis, and heart failure (10–12). Martinet *et al* (13) previously provided evidence of elevated levels of PARP1 in human atherosclerotic plaques. In addition, PARP1 inhibition has been demonstrated to provide protection against endothelial dysfunction in shock, hypertension, and heart failure (14).

iNOS is a critical member among the inflammatory cytokines regulated by PARP1 through the NF-κB pathway. Induction of iNOS results in excessive production of nitric oxide (NO), which reacts with superoxide anions to form peroxynitrite. The production of peroxynitrite results in tyrosine nitration, DNA damage, and activation of PARP1, which leads to changes in inflammatory responses, and promotion of cell death by apoptosis and necrosis (11,15). Numerous studies have demonstrated that neutralization of peroxynitrite is an effective therapeutic against cardiovascular, inflammatory, and neurodegenerative diseases, by providing protection against cell death and downregulating inflammatory responses (15).

In the present study, it was hypothesized that the oxidative DNA damage, that results from the generation of reactive species during the onset of MI, may cause excessive activation of PARP1. Excessive PARP1 may result in an increased expression of iNOS, and an imbalance of cell survival mechanisms that contribute to the death of cardiomyocytes and aggravation of cardiac functions. It may be hypothesized that pharmacological inhibition of PARP1 or iNOS may protect cardiomyocytes from death, and improve cardiac function. A rat model of MI was used to investigate the potential role of PARP1 and iNOS in the process of MI, and to examine the protective effects of their inhibition.

## Materials and methods

### Animals and surgery

A total of 40 male Wistar rats (Animal Experiment Center of Shandong University, Jinan, China), 4 months old, were housed and bred in a pathogen-free animal care facility at the Key Laboratory of Cardiovascular Remodeling and Function Research (Jinan, China). All of the rats were allowed full access to standard mouse chow and water. All experiments were performed in compliance with the Guide for the Care and Use of Laboratory Animals, published by the US National Institutes of Health (NIH Publication No. 85-23, revised 1985; NIH, Bethesda, MA, USA) and Shandong University (Jinan, China).

The rats were anesthetized for a sham operation with sodium pentobarbital (50 mg/kg). In order to induce an MI, the left side of the chest was opened and the left anterior descending coronary artery was occluded by ligation, using a 6-0 polypropylene monofilament suture at the left atrial apex, as described by previous methods (16). Immediately following coronary ligation, all of the rats received a single abdominal injection of either dimethylsulfoxide (DMSO; 100 μl; n=7), 3,4-dihydro-5-[4-(1-piperidinyl)butoxy]-1(2H)-isoquinolinone (DPQ; 10 mg/kg; n=8) (14), or N-(1-naphthyl)ethylenediamine dihydrochloride (1400W; 10 mg/kg; n=8) (17). Both DPQ and 1400W were dissolved in 100 μl DMSO.

### Measurement of cardiac function

The measurement was performed as previously described (18). At three days, and at two and four weeks following MI, the left ventricular (LV) dimension and function were assessed by 2-D transthoracic echocardiography on the isoflurane-anesthetized rats. A 12.5 mHz linear-array probe was used, which has been specifically designed for rat cardiac ultrasonic studies, with an HP Sonos 7500 Imaging System (Philips Medical Systems, Andover, MA, USA). LV end-systolic dimension (ESD) and end-diastolic dimension (EDD) were measured from the short-axis view of the LV at the papillary muscle level. Fractional shortening (FS) was used as a sensitive marker of systolic function, and was calculated using the following equation: FS (%) = ((EDD-ESD)/EDD) × 100. All of the measurements were averaged on three consecutive cardiac cycles, and analyzed by two independent researchers. The mice were humanely euthanized under anesthesia.

### Terminal deoxynucleotidyl-transferase-mediated dUTP nick end labeling (TUNEL)

Four weeks following MI, the rats were sacrificed and the hearts were harvested, fixed in 4% paraformaldehyde, embedded in paraffin, and cut into 5 μm sections. A TUNEL assay was used to evaluate apoptotic activity. A commercially available apoptosis detection kit (Roche Diagnostics GmbH, Mannheim, Germany) was used. The terminal deoxynucleotidyl transferase (TdT) reaction was carried out for 1 h at 37°C, and then 3,3′-diaminobenzidine (DAB) chromogen was applied to the samples. Hematoxylin was used as a counterstain. Myocardial apoptosis was assessed in the area at risk (AAR), as described previously (16). Briefly, in each section, the number of cardiomyocytes and the number of TUNEL-positive cardiomyocyte nuclei, were analyzed under a bright field microscope (Olympus Corporation, Tokyo, Japan). Image-Pro Plus v6.0 (Media Cybernetics, Inc., Rockville, MD, USA) was used to quantify the apoptotic activity, by randomly counting 10 fields of the section, at ×400 magnification.

### Caspase-3 activity assay

Caspase-3 activity was measured using a commercial kit, according to the manufacturer’s instructions (Beyotime Institute of Biotechnology, Haimen, China). In the presence of caspase-3, acetyl-Asp-Glu-Val-Asp p-nitroanilide (Ac-DEVD-pNA) is hydrolysed to the yellow product, p-nitroaniline(pNA). The concentration of pNA was measured at 405 nm, using a spectrophotometer (Shanghai Optical Instrument Factory, Shanghai, China).

### Western blot analysis

The proteins were extracted from the frozen cardiac tissue of each group using radioimmunoprecipitation assay lysis buffer (Beyotime Institute of Biotechnology). The protein samples were quantified with a BCA Protein Assay kit (Beyotime Institute of Biotechnology, Shanghai, China). Equal amounts of protein (50 μg) were loaded onto 10% SDS-PAGE gels and separated by electrophoresis. The blots were then transferred to nitrocellulose membranes (Bio-Rad Laboratories, Inc., Hercules, CA, USA). The membranes were blocked at room temperature with 5% nonfat milk, in Tris-buffered saline containing 0.1% Tween^®^ 20 (TBST), and then incubated at 4°C overnight with an appropriate primary antibody: Rabbit anti-β-actin (1:2,000 dilution; Santa Cruz Biotechnology, Dallas, TX, USA), rabbit anti-cleaved caspase-3 (1:1,000 dilution; Cell Signaling Technology, Danvers, MA, USA), rabbit anti-cleaved PARP (1:500 dilution; Abcam, Cambridge, MA, USA), or rabbit anti-iNOS (1:200 dilution; Santa Cruz Biotechnology). The membranes were then washed with TBST and incubated with horseradish peroxidase (HRP)-conjugated goat anti-rabbit secondary antibody (1:5,000 dilution; Jingmei Biotech Co., Ltd., Shenzhen, China) for 2 h at room temperature. Following a further three washes with TBST, the blots were visualized using Enhanced Chemiluminescence Plus reagents (EMD Millipore, Billerica, MA, USA).

### Immunofluorescence

The heart tissues were fixed in 4% polyformaldehyde overnight and stored at −4°C. Serial cryosections (4–5 μm) embedded in OCT compound were made by using a paraffin slicing machine. The serial cryosections (6 μm) of the infarct-related area (IRA) were permeabilized in phosphate-buffered saline (PBS), containing 0.5% Triton X-100. After being blocked with normal goat serum, the cryosections were incubated with the following primary antibodies, overnight at 4°C: Rabbit anti-PAR antibody (1:200 dilution; Santa Cruz Biotechnology), or rabbit anti-3NT (1:500 dilution; Abcam). The cryosections were then incubated with a HRP conjugated secondary antibody (1:5,000 dilution; Jingmei Biotech Co., Ltd., Shenzhen, China). As P3L2.. A small amount of Prolong Gold antifade reagent, with 4′, 6-diamidino-2-phenylindole (DAPI; Invitrogen Life Technologies, Carlsbad, CA, USA) was used to seal the coverslips. The immunofluorescent images were captured using a P3L21 laser scanning confocal microscope (LSM710; Carl Zeiss AG, Oberchoken, Germany) and analyzed using Image Pro Plus 6.0 (Media Cybernetics, Inc., Rockville, MD, USA).

### Superoxide anion (O_2_^−^) production assay

Following treatment, the IRA of the cardiac tissues were incubated with a fluorescent probe of O_2_^−^, dihydroethidium (DHE), for 30 min at 37°C. The IRA samples were washed with PBS, and the fluorescent signals were measured at 535 nm, using a laser scanning confocal microscope. The fluorescence intensity was measured for ≥10 fields per sample. Three independent experiments were performed.

### Statistical analyses

All of the data are expressed as the means ± standard deviation. SPSS for Windows version 16.0 (SPSS Inc, Chicago, IL, USA) was used for statistical analyses. Intergroup comparisons were performed using a two tailed student’s t test or a one-way analysis of variance, followed by a test of least significant difference (when equal variances were assumed) or a Dunnett’s T3 (when equal variances were not assumed). A P<0.05 was considered to indicate a statistically significant difference.

## Results

Three rats in the MI group and two rats from both the DPQ and 1400W groups succumbed to natural causes. Perioperative mortality (<24 hours following coronary ligation) ranged between 20–30%, and was not affected by the surgery or the dose of the drugs. There was no mortality following the first 24 hours, up to the end of the experiment (4 weeks).

### Inhibition of PARP1 and iNOS activity improves cardiac function following MI

**Figure 1:**
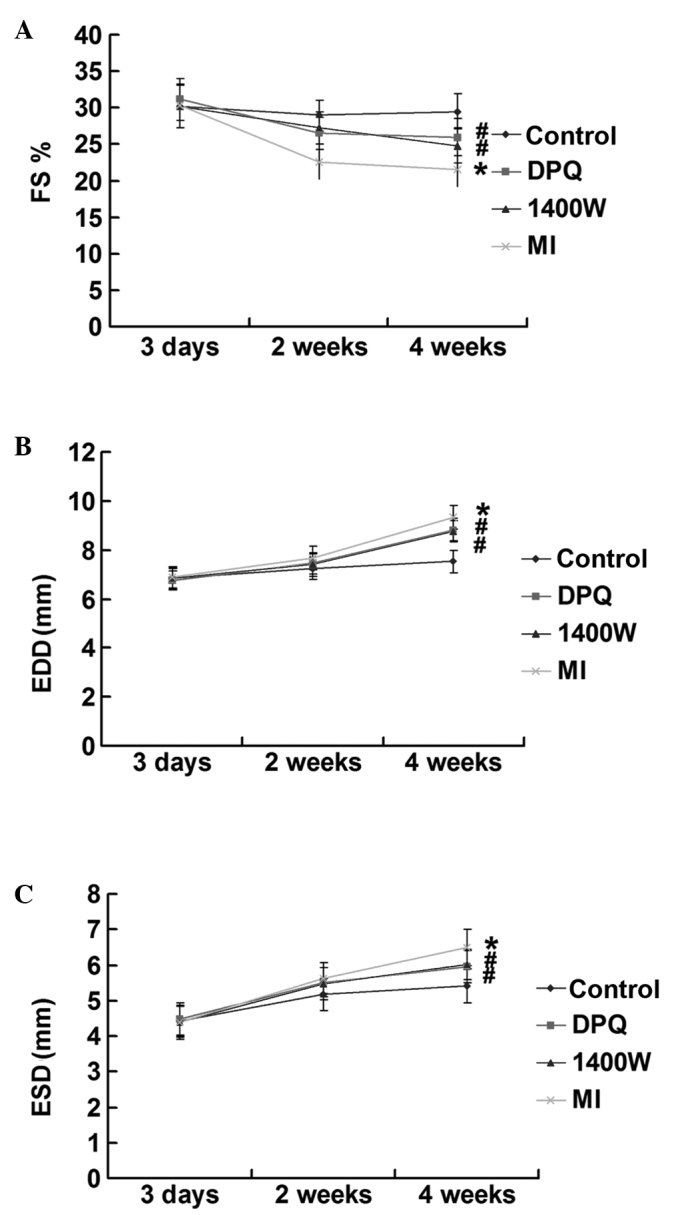
Evaluation of left ventricular dimension and function by echocardiography. (A) Fractional shortening (FS), (B) end-diastolic diameter (EDD), and (C) end-systolic diameter (ESD) of the four groups following myocardial infarction (MI). FS was decreased, and EDD and ESD were increased following MI, ^*^P<0.05 vs the control group; ^#^P<0.05 vs the MI group. DPQ, 3,4-dihydro-5-[4-(1-piperidinyl)butoxy]-1(2H)- isoquinolinone; 1400W, N-(1-naphthyl)ethylenediamine dihydrochloride.

During the four weeks following coronary ligation, the cardiac function of the rats was observed by serial echocardiography, and the FS, LV EDD and ESD were determined (Fig. 1A–C). The control group maintained consistent cardiac function, during the four weeks. In the MI group, the EDD and ESD increased by 19.30 and 16.65%, respectively, four weeks after surgery, and the FS decreased by 26.87%, as compared with the control group (P<0.05). The echo indices of FS and LV enlargement in the DPQ and 1400W groups, following MI, were lower as compared with the control group; however, the reduction was less severe, as compared with the MI group (P<0.05; Fig. 1A–C).

### Inhibition of PARP1 and iNOS activity reduces MI-induced apoptosis

**Figure 2:**
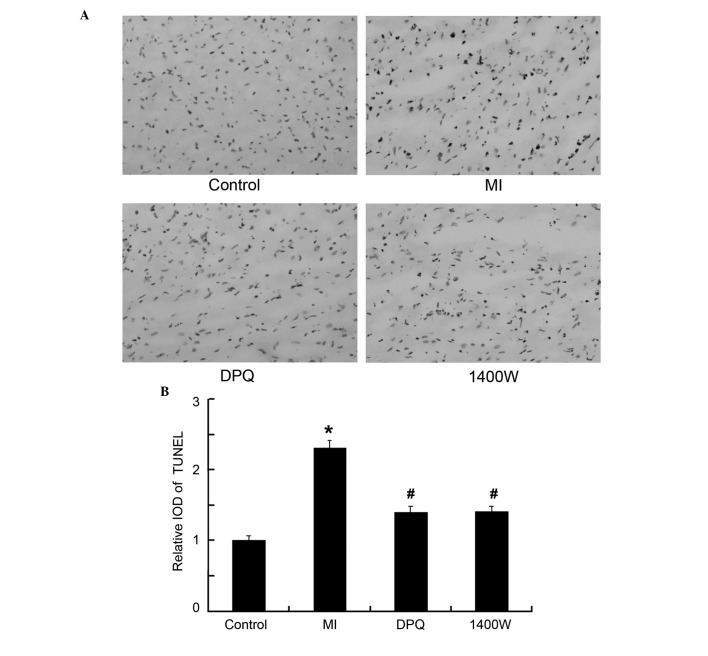
Terminal deoxynucleotidyl-transferase-mediated dUTP nick end labeling (TUNEL) positive cardiomyocytes in the myocardium, four weeks following myocardial infarction (MI). (A) Representative examples of TUNEL staining in myocardium (3,3′-diaminobenzidine staining; magnification, ×400). (B) Percentage of TUNEL-positive cardiomyocytes in the myocardium. Control (n=10); MI (n=7); DPQ (n=8); 1400W (n=8). ^*^P<0.05 vs the control group; ^#^P<0.05 vs the MI group. DPQ, 3,4-dihydro-5-[4-(1-piperidinyl)butoxy]-1(2H)- isoquinolinone; 1400W, N-(1-naphthyl)ethylenediamine dihydrochloride; IOD, integral optical density.

The rate of apoptosis was measured histologically by TUNEL assay (Fig. 2A). The average integral optical density (IOD) for TUNEL-positive cardiomyocytes within the myocardial AAR was significantly increased in the MI group, as compared with the control group (4.05±0.41 vs 1.75±0.12, P<0.05; Fig. 2B). The rate of apoptosis was significantly reduced, by 39.71 and by 39.00% in the DPQ and 1400W treatment groups, respectively, as compared with the MI group (P<0.05, Fig. 2B). There were no significant differences between the rate of apoptosis in the DPQ and 1400W groups.

### Inhibition of PARP1 and iNOS attenuates apoptosis in the AAR by regulating the expression levels of cleaved caspase-3 and PARP1

**Figure 3:**
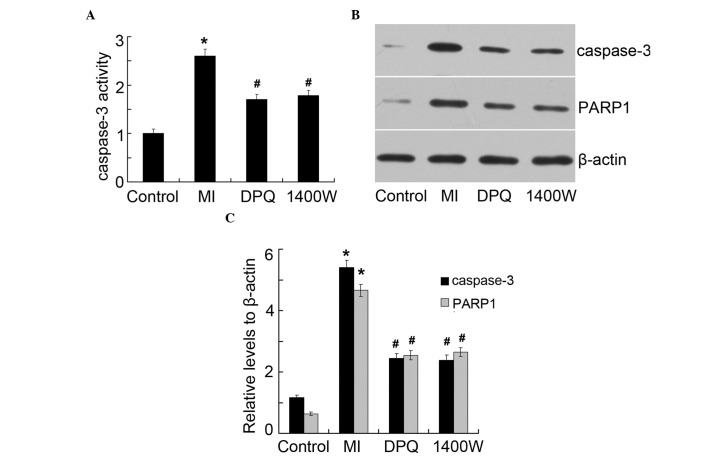
The protein expression levels of cleaved caspase-3 and cleaved poly (ADP-ribose) polymerase (PARP). (A) Caspase-3 activity of cardiomyocytes in the various groups. (B) The protein expression levels of cleaved caspase-3 and cleaved PARP, as determined by western blot analysis. (C) Quantitative analysis of cleaved caspase-3 and cleaved PARP, normalized to β-actin. ^*^P<0.05 vs the control group; ^#^P<0.05 vs the MI group. DPQ, 3,4-dihydro-5-[4-(1-piperidinyl)butoxy]-1(2H)- isoquinolinone; 1400W, N-(1-naphthyl)ethylenediamine dihydrochloride. MI, myocardial infarction.

The main functions of caspase-3 and PARP1 are in apoptosis. The protein expression levels of caspase-3 and PARP1 were examined (Fig. 3). The activity of caspase-3 was 2.60-fold higher in the MI group, as compared with the control group (P<0.05, Fig. 3A). Treatment with DPQ or 1400W significantly decreased caspase-3 activity, as compared with the MI group (P<0.05, Fig. 3A). There was no difference in caspase-3 activity between the two treatment groups. Similar results were determined by western blot analysis (Fig. 3B), and the enhancement of cleaved caspase-3 and PARP1 induced by MI was attenuated by treatment with DPQ or 1400W (P<0.05; Fig. 3C). These results were concordant with the rate of apoptosis detected in each of the groups.

### DPQ and 1400W effectively inhibit MI-induced PARP1 and iNOS activity

**Figure 4:**
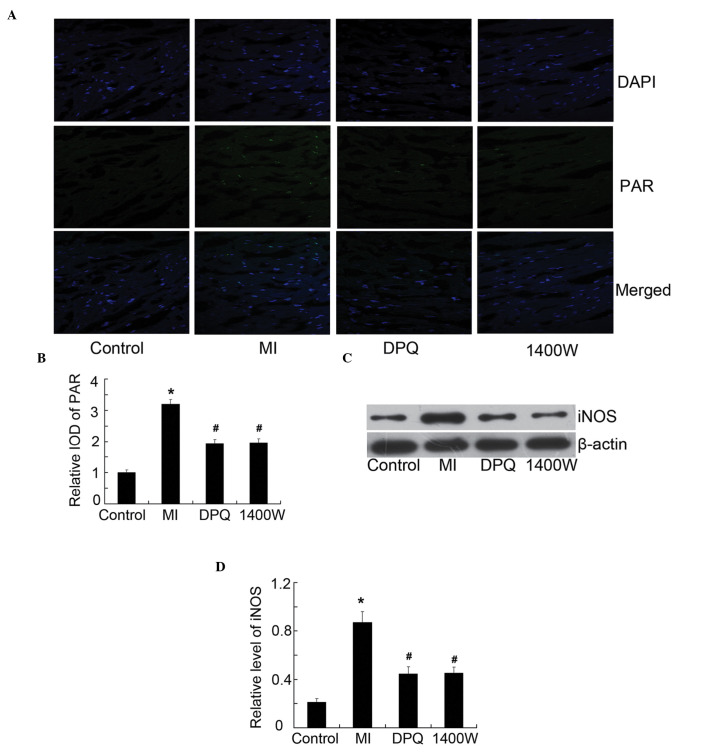
The protein expression levels of poly (ADP-ribose) (PAR) (active PAR polymerase), and inducible nitric oxide synthase (iNOS), in the myocardium of the various groups. (A) Representative fluorescent images of the myocardium, acquired by laser scanning confocal microscopy (magnification, ×400); PAR staining is green, and 4′, 6-diamidino-2-phenylindole (DAPI) staining for the cell nuclei is blue. (B) Quantitative analysis of PAR, expressed as a fold increase over the control group. (C) The protein expression levels of iNOS were examined by western blot analysis, with the indicated antibody. (D) Quantitative analysis of iNOS, relative to β-actin. Control, n=10; MI, n=7; DPQ, n=8; 1400W, n=8.*P<0.05 vs the control group; #P<0.05 vs the MI group. DPQ, 3,4-dihydro-5-[4-(1-piperidinyl)butoxy]-1(2H)- isoquinolinone; 1400W, N-(1-naphthyl)ethylenediamine dihydrochloride; IOD, integral optical density. MI, myocardial infarction.

In order to verify the respective inhibition efficiency of DPQ and 1400W on PARP1 and iNOS, the expression levels of PAR (active PARP) and iNOS were detected in the myocardium of the various groups (Fig. 4). The activity of PAR was detected by immunofluorescence (Fig. 4A). The average IOD was enhanced 3.20-fold in the MI group, as compared with the control (P<0.05, Fig. 4B); however, treatment with DPQ or 1400W reduced MI-induced PAR activity by 39.38 and 38.75%, respectively (P<0.05; Fig. 4B). Simultaneously, the expression levels of iNOS were enhanced 4.14-fold following MI, as compared with the control group, as determined by western blotting (P<0.05, Fig. 4C and D). The inhibition efficiency of DPQ and 1400W was 51.14 and 51.84% respectively, as compared with the MI group (P<0.05, Fig. 4C and D). Notably, DPQ and 1400W could interact with each other in the inhibition of PARP1 and iNOS.

### Inhibition of PARP1 and iNOS repressed O_2_^−^ and nitrotyrosine (3-NT), induced by MI

**Figure 5:**
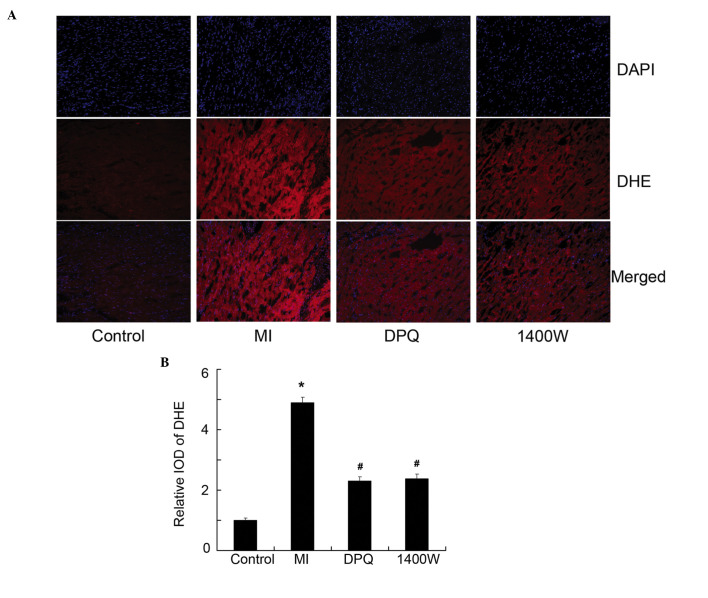
The expression of O_2_^−^ detected by dihydroethidium (DHE). (A) The representative images of O_2_^−^ detected by DHE (red) in the myocardium, 4′, 6-diamidino-2-phenylindole (DAPI) staining for the cell nuclei is blue. (B) Quantitative analysis of DHE, expressed as a fold increase over the control group. Control, n=10; MI, n=7; DPQ, n=8; 1400W, n=8. ^*^P<0.05 vs the control group; ^#^P<0.05 vs the MI group. DPQ, 3,4-dihydro-5-[4-(1-piperidinyl)butoxy]-1(2H)- isoquinolinone; 1400W, N-(1-naphthyl)ethylenediamine dihydrochloride; IOD, integral optical density. MI, myocardial infarction.

**Figure 6:**
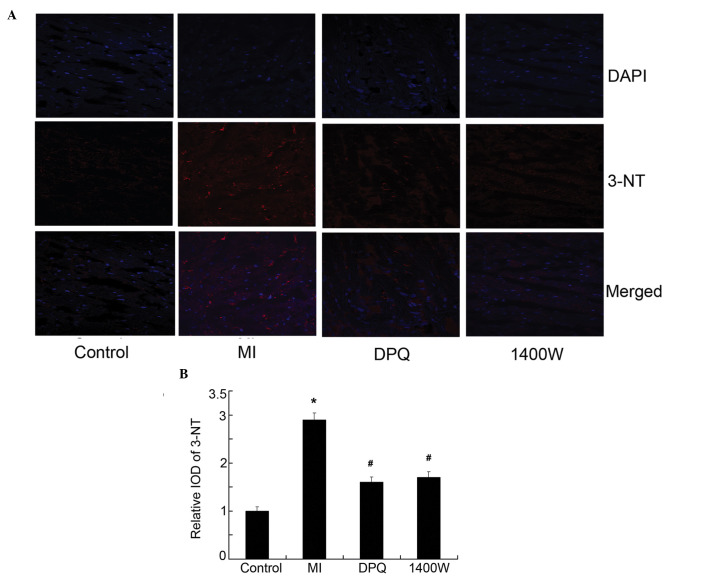
The relative levels of nitrotyrosine (3-NT) in the myocardium. (A) Fluorescent micrographs showing the relative level of 3-NT (red) in the myocardium, 4′, 6-diamidino-2-phenylindole (DAPI)staining for the cell nuclei is blue. (B) Quantitative analysis of 3-NT, expressed as a fold increase over the control group. Control, n=10; MI, n=7; DPQ, n=8; 1400W, n=8. ^*^P<0.05 vs the control group; ^#^P<0.05 vs the MI group. DPQ, 3,4-dihydro-5-[4-(1-piperidinyl)butoxy]-1(2H)- isoquinolinone; 1400W, N-(1-naphthyl)ethylenediamine dihydrochloride; IOD, integral optical density. MI, myocardial infarction.

To further elucidate the signaling pathways involved in MI-induced apoptosis, the expression of O_2_^−^ and the production of NO (3-NT) was determined. The production of intracellular O_2_^−^ was significantly increased in the MI group, as compared with the control group, as determined by DHE detection (P<0.05, Fig. 5A and B). Similarly, the expression of 3-NT was significantly higher in the MI group, as compared with the control group, as determined by immunofluorescence (P<0.05, Figure 6A, B). Treatment with DPQ or 1400W effectively reversed the high O_2_^−^ levels induced by MI, by 53.07 and 51.43% (<0.05, Fig. 5A and B), and reduced 3-NT expression by 44.87 and 41.38% (P<0.05, Fig. 6A and B), respectively.

## Discussion

The present study tested the hypothesis that PARP1 and iNOS contribute to the deleterious effects on cardiac function, exerted by cardiomyocyte apoptosis, following MI. PARP1 and iNOS were shown to be upregulated, alongside an increased rate of caspase-3-dependent apoptosis, and deterioration of cardiac function in rats, following an MI. The PARP inhibitor DPQ and the iNOS inhibitor 1400W attenuated these effects. Furthermore, the reduction in the rate of apoptosis, by DPQ and 1400W, was associated with the reduced production of O_2_^−^ and 3-NT in the ischemic myocardium.

Previous studies have evaluated cardiac function following induction of MI by serial echocardiography (2,19), and demonstrated that it is associated with apoptosis and inflammation in the AAR (20). In concordance with previous reports, the results of the present study showed that apoptosis following MI and production of iNOS were markedly elevated, with deterioration of cardiac function. Considering the involvement of PARP1 in various cardiovascular and inflammatory diseases, these results emphasize its role in the pathogenesis of MI.

Previous evidence has suggested that PARP may be excessively activated by reactive oxygen and nitrogen species in cardiomyocytes and endothelial cells, during myocardial ischemia/reperfusion injury, various forms of heart failure or cardiomyopathies, circulatory shock, cardiovascular aging, diabetic complications, myocardial hypertrophy, atherosclerosis, and vascular remodeling following injury (21). Furthermore, severe DNA damage may lead to initiation of the second apoptotic pathway, in which caspases inactivate PARP1, by cleaving it into two fragments, therefore preventing it from responding to DNA strand breaks (22,23). Reactive oxygen species, which have been implicated in cardiac pathophysiology, can trigger apoptosis of myocytes by upregulating pro-apoptotic proteins, such as Bcl-2-associated X protein and caspases, and the mitochondria-dependent pathway (24). The results of the present study indicated that inhibition of PARP1 with DPQ, could reduce O_2_^−^ and ONOO^−^ in rats following MI, and reduce caspase-3-dependent apoptosis. These results were concordant with previous findings that the PARP1 inhibitor PJ34 limited myocardial damage, due to post-ischemic reperfusion, by decreasing NAD^+^ and ATP, and the caspase-dependent pathway (25).

Another role of PARP1 is its regulation of inflammation at the transcriptional level (e.g., iNOS, intracellular adhesion molecule-1, cyclooxygenase-2). The absence of functional PARP-1 has been shown to decrease the expression levels of proinflammatory mediators, including cytokines, chemokines, adhesion molecules and enzymes, such as iNOS (26). Previous results have suggested that iNOS expression contributes to PARP activation in cerebral ischemia, and a previously unrecognized deleterious interaction between iNOS and PARP has been identified (27). The present study demonstrated that inhibition of PARP1 reduced the expression of iNOS, meanwhile the inhibitor of iNOS, 1400W, attenuated the expression of PARP1 and resulted in the reduction of apoptosis. The interaction between PARP1 and iNOS requires further study.

iNOS is one of the most important NO donors, and is associated with numerous important pathophysiological processes in various conditions, including MI and heart failure (28,29). Cell apoptosis and NT formation have previously been attenuated by selective iNOS inhibitors, or in iNOS knockout mice (30). Peroxynitrite is a reactive oxidant which is formed by reaction of NO with the superoxide anion (11). ONOO^−^ has been shown to function as an important trigger of myocardial necrosis and apoptosis in various cardiac pathologies, by inducing oxidative DNA damage, which may lead to the activation of the DNA repair enzyme PARP. PARP subsequently consumes cellular NAD^+^, ultimately leading to ATP depletion and necrotic cell death (21,31,32). The results of the present study showed that the iNOS inhibitor, 1400W, could attenuate NT formation and apoptosis, and could reduce the activity of PARP1.

In conclusion, the present study provided novel evidence that pharmacological inhibition of PARP1 with DPQ and iNOS with 1400W, improved cardiac function in rats, following MI, by attenuating cell apoptosis and inflammation. The predicted pathway of these effects is that DPQ may downregulate the inflammatory factor iNOS and its effects on O_2_^−^ and ONOO^−^ in MI rats, and reduce the rate of caspase-3-dependent apoptosis. In addition, inhibition of iNOS decreased the levels of reactive oxygen and nitrogen species, resulting in the reduction of DNA single strand breaks and PARP1 activiation, leading to similar protection in ischemic myocardium. The effects of PARP1 and iNOS inhibitors may be exploited for the experimental therapy of disease.
